# Identification of Circulating Serum Multi-MicroRNA Signatures in Human DLBCL Models

**DOI:** 10.1038/s41598-019-52985-x

**Published:** 2019-11-20

**Authors:** Afshin Beheshti, Kristen Stevenson, Charles Vanderburg, Dashnamoorthy Ravi, J. Tyson McDonald, Amanda L. Christie, Kay Shigemori, Hallie Jester, David M. Weinstock, Andrew M. Evens

**Affiliations:** 1 WYLE, NASA Ames Research Center, Moffett Field, CA USA; 2 Division of Blood Disorders, Rutgers Cancer Institute of New Jersey, New Brunswick, NJ USA; 3 Department of Medical Oncology, Dana-Farber Cancer Institute, Boston, MA USA; 4 Harvard Medical School, Boston, MA USA; 5 Stanley Center for Psychiatric Research, Broad Institute of MIT and Harvard, Cambridge, MA USA; 6 Cancer Research Center and Department of Physics, Hampton University, Virginia, USA

**Keywords:** Cancer, Non-hodgkin lymphoma, Tumour biomarkers, miRNAs, Diagnostic markers

## Abstract

There remains a need to identify new sensitive diagnostic and predictive blood-based platforms in lymphoma. We previously discovered a novel circulating microRNA (miRNA) signature in a Smurf2-deficient mouse model that spontaneously develops diffuse large B-cell lymphoma (DLBCL). Herein, we investigated this 10-miRNA signature (miR-15a, let-7c, let-7b, miR-27a, miR-10b, miR-18a, miR-497, miR-130a, miR24, and miR-155) in human lymphoma cell lines, mice engrafted with patient-derived xenografts (PDXs), and DLBCL patient serum samples leveraging systems biology analyses and droplet digital PCR (ddPCR) technology. Overall, 90% of the miRNAs were enriched in PDX DLBCL models and human lymphoma cell lines. Circulating miRNAs from the serum of 86 DLBCL patients were significantly increased compared with healthy controls and had similar patterns to the murine models. Strikingly, miRNAs were identified up to 27-fold higher levels in the serum of PDX-bearing mice and human patients compared with lymphoma cell lysates, suggesting a concentration of these factors over time within sera. Using cut-points from recursive partitioning analysis, we derived a 5-miRNA signature (let-7b, let-7c, miR-18a, miR-24, and miR-15a) with a classification rate of 91% for serum from patients with DLBCL versus normal controls. In addition, higher levels of circulating let-7b miRNA were associated with more advanced stage disease (i.e., III-IV vs. I-II) in DLBCL patients and higher levels of miR-27a and miR-24 were associated with *MYC* rearrangement. Taken together, circulating multi-miRNAs were readily detectable in pre-clinical cell line and human lymphoma models as well as in DLBCL patients where they appeared to distinguish clinico-pathologic subtypes and disease features.

## Introduction

Diffuse large B cell lymphoma (DLBCL) is the most common non-Hodgkin lymphoma (NHL) in the United States and worldwide^1,2^. Although 50–60% of DLBCL patients are cured with multi-agent chemoimmunotherapy^1,2^, a significant fraction of patients relapse and die from this malignancy^3,4^. Higher-risk populations of DLBCL have been identified, such as the 20–25% of patients with high protein expression of Myc and Bcl2 as well as 5–10% of patients with *MYC* gene rearrangements^5,6^. Recent studies have identified additional risk subsets based on mutations, copy number alterations and gene expression profiling^5,7^, but these are primarily based on tumor biopsies/specimens and it remains unclear how to optimally integrate into clinical practice. Early data using serum analytes such as circulating tumor DNA^8^ suggest that”blood biopsy” approaches may be tractable for diagnosing DLBCL and even distinguishing subtypes and therapeutic responses.

MicroRNAs (miRNA) are small, non-coding RNA of approximately 22 nucleotides that target hundreds of messenger RNAs, regulate proteins, and interact with DNA^9–13^. Due to their small size, circulating miRNAs are stable and resistant to degradation^14,15^. Most literature linking miRNAs and lymphoma have concentrated on one or two different miRNAs that could serve as biomarkers^9–12,16,17^ or single miRNA-related treatments^16,17^. In 2008, Lawrie *et al*. reported associations involving three circulating miRNAs in cases of DLBCL^18^. We hypothesized that a more expansive multi-miRNA signature assessment could define combinations of miRNA that distinguish DLBCL state, prognosis and/or therapeutic response.

Using a Smurf2-deficient mouse model that spontaneously develops DLBCL^19,20^, we previously demonstrated that a circulating miRNA signature consisting of 10 miRNAs (miR-15a, let-7c, let-7b, miR-27a, miR-10b, miR-18a, miR-497, miR-130a, miR24, and miR-155) was detectable in the serum 12 months prior to symptoms of tumor formation^19^. In this study, we determined the DLBCL miRNA signature by focusing on the miRNAs that were conserved between the serum and different tissues (i.e. bone marrow and tumor). For validation, we utilized digital-droplet PCR (ddPCR) for ultrasensitive (i.e., <1 molecule/ng of serum RNA) detection and quantification of miRNAs in serum.

Herein, we investigated this novel 10-miRNA signature in the tissue and serum from patient-derived xenograft (PDX) models of DLBCL^21^, DLBCL cell lines, and from serum samples of DLBCL patients at multiple time points before, during, and after therapy. Furthermore, we sought to correlate circulating miRNA serum signatures with disease characteristics as well as patient clinical status (i.e., length of remission).

## Results

### DLBCL PDX models and DLBCL cell lines express high amounts of miRNA

**Figure 1:**
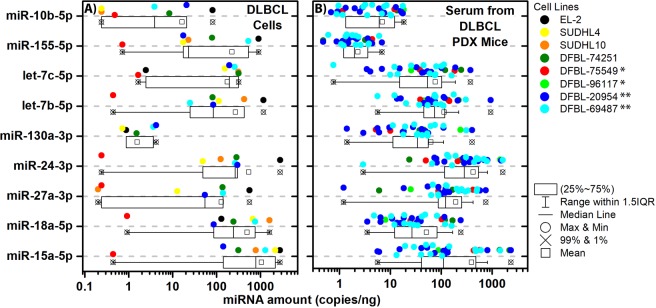
The amounts of miRNA in the DLBCL cell lines and PDX murine models. (**A**) Boxplot showing the amount of miRNA (copies/ng) for each miRNA part of the miRNA signature that are contained in commercial DLBCL cell lines (SUDHL4 and SUDHL10), primary human lymphoma cells isolated from discarded tumor biopsies (EL-2), and PDX cell lines (DFBL-74251, DFBL-69487, DFBL-75549, and DFBL-20954). Each cell line represents a biological replicate for DLBCL for a total of n = 7 DLBCL cell lines. (**B**) Box plots of the amount of miRNA (copies/ng) quantified from the serum of PDX mice implanted with the following PDX cell lines: DFBL-74251 (n = 2), DFBL-69487 (n = 13), DFBL-75549 (n = 2), DFBL-20954 (n = 13), and DFBL-96117 (n = 2). Single MYC + (*) and MYC/BCL2 Double-Hit (**) DLBCL cells are designated in the figure legend next to the cell names. In the boxplots the whiskers show the range of the outliers, with maximum and minimum values as “o” and the 1st and 99^th^ percentile outliers as “X”, the mean values are shown as “□”, and the median line is shown as “—”. The data points for each sample is shown on the top of the boxplots and are color coded to match the specific cell lines.

Using the previously discovered circulating 10 multi-miRNA signature (i.e., miR-15a, let-7c, let-7b, miR-27a, miR-10b, miR-18a, miR-130a, miR-24, miR-155, and miR-497), we quantified miRNA levels across two human DLBCL cell lines (SUDHL4 and SUDHL10) and a culture of a primary/fresh human DLBCL cells (EL-2), the latter isolated from a patient tumor with circulating cancer cells. We also examined the presence of this miRNA signature from the serum of five DLBCL PDX models. It is important to highlight that the sequences for the miRNAs are conserved between mouse and human for the miRNAs listed above. All miRNAs, except for miR-497, were highly elevated in the DLBCL cells *in vitro* (Fig. 1A) when comparing to our older findings from our previous publication^19^. In our previous analysis of Smurf2-deficient mice^19^, miR-497 also had the lowest amount of change from controls. Due to this, we excluded it from the miRNA signature.

For the mice engrafted with the DLBCL PDX models, we examined five models with or without *MYC* and *BCL2* molecular re-arrangements: DFBL-74251 (*BCL2* rearrangement only), DFBL-75549 and DFBL-96117 (*MYC* rearrangement only), and DFBL-69487 and DFBL-20954 (*MYC/BCL2* double-hit). The concentrations of the miRNAs circulating in the serum of PDX-bearing mice ranged from 0.01 to 27.37 fold-change compared with the original PDX cell line (Fig. 1B and Supplemental Table S1), with the exception of one PDX cell line (DFBL-75549) that had 10x to 1000x more miRNA present in the serum of mice versus the original PDX cell line. The actual circulating miRNA concentrations in the serum of PDX-bearing mice ranged from 0.1 to 584.0 ng/copies (Fig. 1B). Surprisingly, this indicated that the presence of the miRNAs were mostly higher in the serum of the PDX-bearing mice than the original cell lines with all cell lines having an increase in 4 or more miRNAs.

### Circulating miRNA in healthy controls compared with DLBCL patients

**Figure 2:**
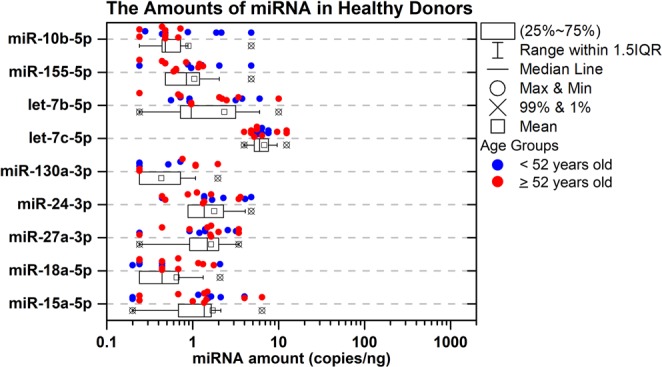
The amount of miRNA in healthy subjects. A boxplot of the amount of miRNA (copies/ng) quantified from the serum of n = 17 healthy subjects. The whiskers show the range of the outliers, with maximum and minimum values as “o” and the 1st and 99^th^ percentile outliers as “X”, the mean values are shown as “□”, and the median line is shown as “—”. The data points for each sample is shown on the top of the boxplots and are color coded for <52 years old as blue and ≥52 years old as red.

To characterize miRNA expression in non-tumor bearing individuals, we isolated miRNA from the serum of 17 healthy controls. The median age of the healthy controls was 52 years (range, 29–70 years) and 65% were female (Supplemental Table S2). In general, the 10 miRNAs within the DLBCL were present at very low amounts in the serum of healthy individuals, with the median value of the miRNAs ranging only between 0.24 to 1.48 copies/ng for 9 of the miRNAs. The one exception was let-7c that had a median value of 6.0 copies/ng (Supplemental Table S3). Marginal differences in miRNA expression for the healthy controls by sex (males vs. females) were noted for miR-155-5p (median 0.4 vs. 1.0; *P* = 0.049) and mir-10b-5p (median 0.6 vs. 0.0; *P* = 0.029) (Fig. 2 and Supplemental Table S3).

We also obtained and analyzed serum from 86 DLBCL patients in various stages of disease treatment (Supplemental Table S2) that included: prior to treatment with first-line therapy (or the initial treatment for the lymphoma) (n = 11); during treatment (also referred to on-treatment) (n = 16); progression after treatment (n = 7); and post-therapy in complete remission (n = 52). Progression after treatment typically can happen at any time, but is most common within the first two years after diagnosis. *MYC* molecular rearrangements were present in 13 (15%) patients; 26 (30%) patients were positive for MYC protein by immunohistochemical staining (IHC) in ≥40% of lymphoma cells. Fifty-two (60%) patients were Bcl2 protein positive and 59 (69%) were BCL6 positive by IHC. Clinically, 40% of patients were stage 3 or 4 at the time of diagnosis and 45% had received 2 or more regimens prior to the time of sample collection. Twenty-six percent of patients had received a prior autologous stem cell transplant (autoSCT), 6 had an allogeneic SCT (alloSCT), and 5 patients had received prior chimeric antigen receptor T cell (CAR-T) therapy (Supplemental Table S2). Of the 52 patients in complete remission (CR), 21 (40%) were in CR for >24 months at the time of sample collection.

Higher circulating levels of let-7b were associated with a higher stage at diagnosis (stage I-II vs III-IV median 54.0 vs. 163.2; *P* = 0.007) and higher levels of both miR-27a and miR-24 were associated with having a *MYC* rearrangement (median 20.0 vs. 4.8; *P* = 0.003 for miR-27a; median 58.4 vs. 24.4; *P* = 0.046 for miR-24) independent of disease state. Higher levels of miR-18a were associated with Myc protein expression (0–9% vs. 10–59% vs. ≥60%, median 3.3 vs. 2.8 vs. 8.8; *P* = 0.03) and not being treatment-naïve (median 4.8 vs. 1.1; *P* = 0.016). Thus, 4 of the 5 miRNAs identified in the signature had an association with *MYC* rearrangement or IHC protein expression.

Patients with DLBCL and controls differed based on two factors. The median age of DLBCL patients was 64 years (range, 21–87) vs. 52 years (range, 29–70) for healthy controls (p < 0.001). Sixty-nine percent of DLBCL patients were male vs. 35% of healthy controls (p = 0.012).

Overall, we identified significantly increased circulating levels of the miRNAs in the serum of DLBCL patients compared with healthy controls (Table 1). Specifically, we observed that miR-24, mir-18a, miR-15a, let-7b were significantly higher (see p-values in Table 1) for patients on-treatment, at progression and in complete remission compared to healthy subjects (two-sided Wilcoxon rank-sum test p < 0.01). In addition, miR-130a and miR-7c were significantly higher for patients on-treatment or in complete remission (p < 0.01) compared to healthy subjects, but only let-7c was significantly higher for those pre-treatment (p < 0.0001). For miRNA-155 and miR-10b there was not a significant increase when comparing healthy to progression patients, although all other conditions were increased.

**Table 1:** Median and Interquartile Range of miRNA Concentration Copies/ng by Treatment/Outcome Group.

N	Healthy conc copies/ng median (IQR)	Pre-Treatment conc copies/ng median (IQR)	On-Treatment conc copies/ng median (IQR)	Progression conc copies/ng median (IQR)	Complete Remission conc copies/ng median (IQR)
	17	11	16	7	52
miR-24	1.4 (0.9, 2.3)	39.8 (0.4, 327.2) P = 0.22	51.4 (9.4, 95.0) P < 0.0001	12.4 (3.1, 90.8) P = 0.008	48.2 (11.4, 157.0) P < 0.0001
miR-18a	0.4 (0.2, 0.7)	1.1 (0.7, 4.4) P = 0.025	6.0 (3.7, 8.2) P < 0.0001	3.7 (2.1, 9.6) P = 0.0003	4.4 (1.8, 10.0) P < 0.0001
miR-15a	1.4 (0.7, 1.6)	8.8 (0.6, 52.0) P = 0.024	23.6 (12.8, 46.6) P < 0.0001	5.2 (4.0, 29.6) P = 0.0009	20.4 (4.7, 43.8) P < 0.0001
let-7c	6.0 (5.2, 7.6)	26.0 (11.2, 78.4) < 0.0001	73.2 (26.4, 115.2) P < 0.0001	22.8 (14.0, 37.6) P = 0.013	35.0 (12.6, 102.0) P = 0.0002
let-7b	1.0 (0.7, 3.2)	198.0 (0.8, 353.2) 0.066	150.2 (84.6, 347.4) P < 0.0001	51.2 (4.4, 155.2) P = 0.002	75.0 (20.2, 247.6) P < 0.0001
miR-27a	1.5 (0.9, 2.0)	1.8 (0.3, 54.8) P = 0.45	5.5 (1.4, 21.4) P = 0.027	2.9 (1.6, 25.2) P = 0.049	12.8 (4.6, 52.2) P < 0.0001
miR-155	0.8 (0.5, 1.2)	3.5 (0.4, 9.2) P = 0.066	2.0 (0.7, 3.5) P = 0.056	0.8 (0.5, 2.0) P = 0.73	1.5 (0.6, 3.6) P = 0.031
miR-130a	0.2 (0.0, 0.7)	2.2 (0.0, 14.0) P = 0.028	8.4 (2.6, 19.2) P < 0.0001	1.4 (0.0, 17.2) P = 0.079	4.2 (1.5, 19.0) P < 0.0001
miR-10b	0.5 (0.4, 0.7)	1.2 (0.2, 1.7) P = 0.36	1.3 (0.8, 2.0) P = 0.040	0.6 (0.4, 1.2) P = 0.46	0.9 (0.4, 2.0) P = 0.17

two-sided Wilcoxon rank-sum test vs. Healthy; IQR = interquartile range.

### Circulating miRNA signatures in DLBCL patients based on disease status

**Figure 3:**
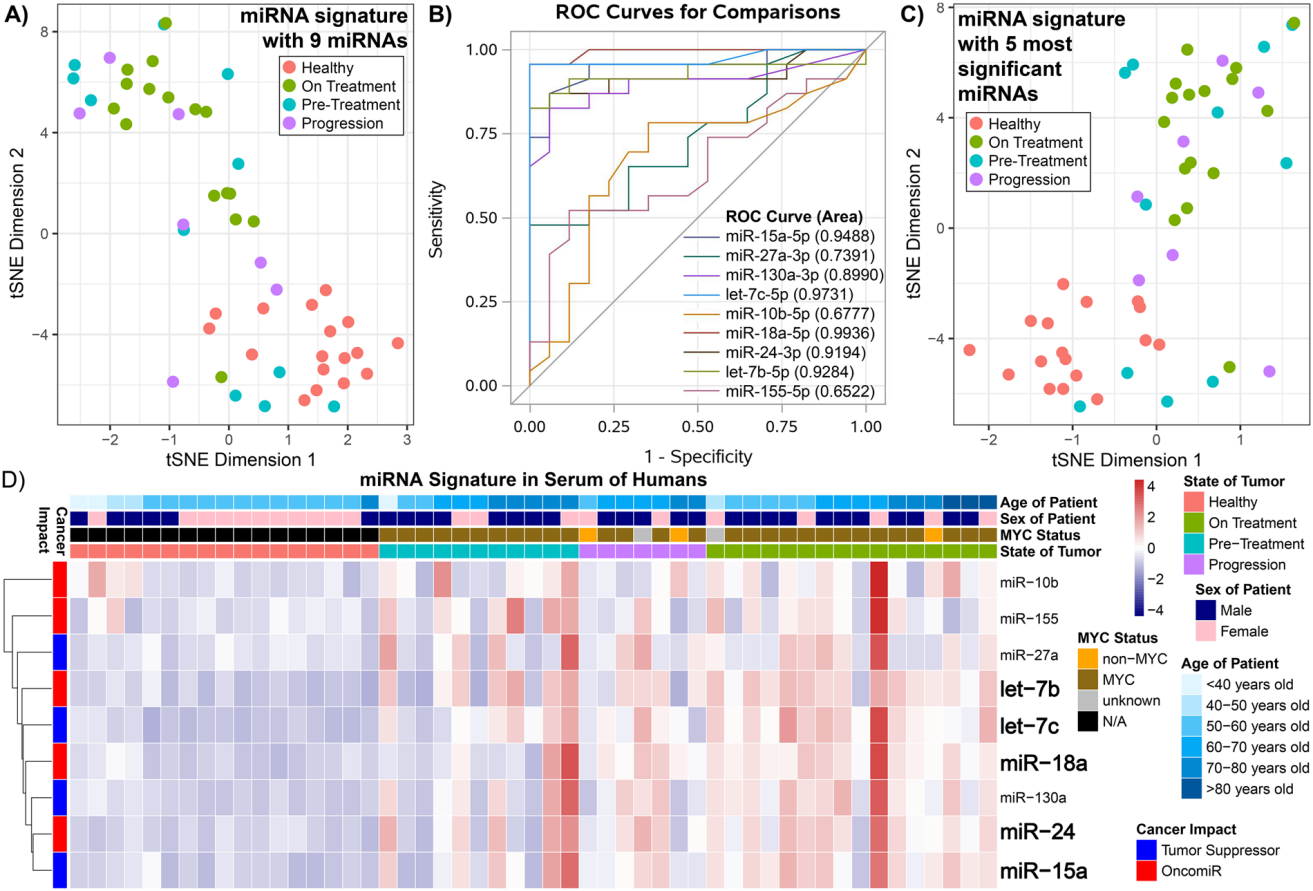
Comparison of the circulating miRNA signature among DLBCL patients and healthy subjects. (**A**) t-Distributed Stochastic Neighbor Embedding (t-SNE) plot showing the distribution of the overall response of the circulating miRNA signature (containing all 9 miRNAs) from the serum between the healthy subjects (red), DLBCL patients pre-treatment (blue), DLBCL patients progression after treatment (purple), and DLBCL patients which are currently on treatment (olive). (**B**) Receiver Operating Curves (ROCs) for the Progression/On-treatment (n = 23) Compared to Healthy (n = 17) Samples. The area under the curve is shown in parentheses. (**C**) t-SNE plot determined by using the 5 most significant miRNAs (Supplemental Table S4 and (**B**)) for the same patient samples as in the previous plot. (**D**) Heatmap representing hierarchical clustering of the circulating miRNA signature. The specific subject information for age, MYC status, state of tumor, and miRNA cancer impact is shown. For all plots the miRNA amounts were log_2_(x + 1) transformed.

We visualized the data using t-Distributed Stochastic Neighbor Embedding (t-SNE) to determine separation between healthy subjects and DLBCL patients (Fig. 3A). Interestingly, 4/11 (36%) patient samples in the pre-treatment group clustered with healthy controls compared to only 3/23 (13%) progression/on-treatment samples.

Based on the t-SNE analysis, we used the 23 on-treatment and progression samples to determine cut-points for receiver operating curve analysis (ROC) (Fig. 3B). Using the Youden Index for each of the 9 miRNAs we were also able to correctly classify the remission samples vs healthy. Sensitivity in distinguishing healthy controls from DLBCL patients samples ranged from 44–85% and specificity ranged from 65–100% for individual miRNAs (Fig. 3B, Supplemental Table S4). Individually, six of the 9 miRNAs had a classification rate ≥80%, with miR-24 performing the best (classification rate 88%, sensitivity 85%, specificity 100%). Using similar cutpoints obtained from recursive partitioning analysis, we derived 5-miRNAs (let-7b, let-7c, miR-18a, miR-24, and miR-15a), which was associated with an improved classification rate of 91% (sensitivity 90% and specificity 94%). Based on this 5 multi-miRNA signature, we established clearer separation in the t-SNE plots between healthy subjects and DLBCL patients (pre-treatment, progression, and on treatment) (Fig. 3C,D).

**Figure 4:**
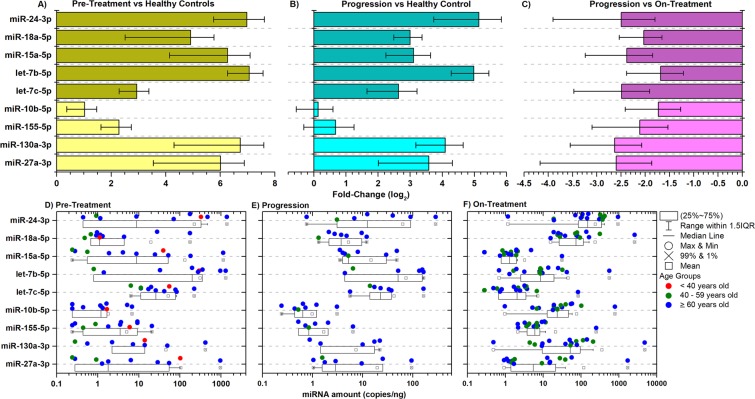
Analysis of circulating miRNAs between healthy subjects and DLBCL patients based on disease status. The log_2_ fold-change values directly comparing (**A**) the average circulating miRNA amounts from the serum between the pre-treatment DLBCL patients versus the healthy controls, (**B**) the progression after treatment DLBCL patients versus the healthy controls and (**C**) the progression after treatment DLBCL patients versus the on-treatment DLBCL patients. The darker shade of the color for (**B**,**C**) and (**D**) represent the 5 most significant miRNAs. Boxplots of the actual amounts of miRNAs (copies/ng) which are present in (**D**) the serum for pre-treatment DLBCL patients, (**E**) progression after treatment in DLBCL patients, and (**F**) on-treatment in DLBCL patients. The whiskers show the range of the outliers, with maximum and minimum values as “o” and the 1st and 99^th^ percentile outliers as “X”, the mean values are shown as “□”, and the median line is shown as “—”. The data points for each sample is shown on the top of the boxplots and are color coded for <40 years old as red, 40–59 years old as olive, and ≥60 years old as blue.

All 9 miRNAs were elevated in pre-treatment DLBCL patient serum samples compared with healthy controls (Fig. 2), of which 5 met statistical significance (*P* ≤ 0.01) (Fig. 4A,D). In addition, these 5 significant miRNAs as noted above (let-7b, let-7c, miR-18a, miR-24, and miR-15a) had the largest fold-changes with the lowest variability between progression and pre-treatment patients (Fig. 4A,B). The actual elevated levels of the miRNAs (copies of miRNAs per ng in the serum) became apparent in DLBCL patients for both pre-treatment (Fig. 4D**)** and progression classification (Fig. 4E) when comparing to the healthy population (Fig. 2). Interestingly, when comparing the levels of the miRNAs between progression and on-treatment patients we observe a decrease in the miRNA levels (Fig. 4C,F). Since progression is defined as progression after treatment, this result can point to the fact that the treatment has some effect on the tumor for the DLBCL patients indicated by the reduction of the circulating miRNA signature.

### DLBCL patient remission status based on multi-miRNA signature

**Figure 5:**
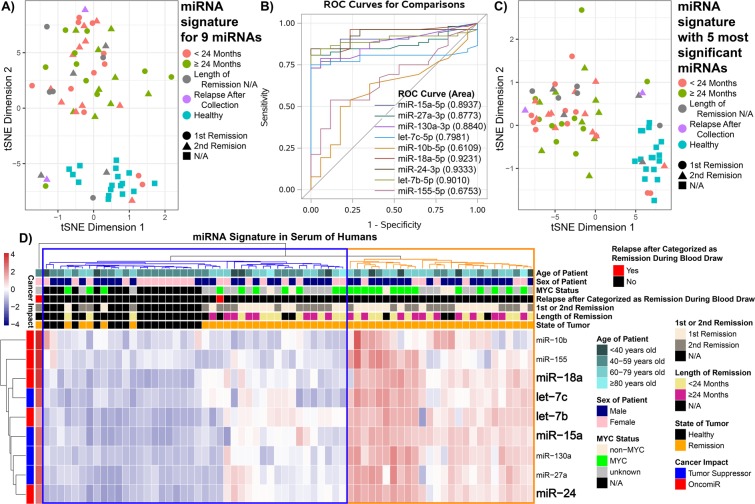
Presence of the miRNA signature for DLBCL patients in remission. (**A**) t-SNE plot showing the distribution of the overall response of the circulating miRNA signature (containing all 9 miRNAs) from the serum between the DLBCL in remission for <24 months (red), DLBCL patients in remission for ≥24 months (olive), DLBCL patients with the length of remission unknown (grey), DLBCL patients which at the time blood collection were categorized as remission, by follow up data indicates that patients relapsed with DLBCL (purple), and healthy subjects (blue). In addition, the DLBCL remission patients were classified as 1^st^ remission (●) or 2^nd^ remission (▲). (**B**) Receiver Operating Curves (ROCs) for the Remission (n = 52) vs. Healthy (n = 17) Samples. The area under the curve is shown in parentheses. (**C**) t-SNA plot determined by using the 5 most significant miRNAs (Supplemental Tables S4 and S5) for the same patient samples as in the previous plot. (**D**) Heatmap representing hierarchical clustering of the circulating miRNA signature. The specific subject information for age, MYC status, state of tumor, length of remission, either 1^st^ or 2^nd^ remission, relapse after categorized as remission during blood draw, and miRNA cancer impact is shown. For all plots the miRNA amounts were log_2_(x + 1) transformed.

Next, we quantified the circulating miRNA signature in the DLBCL patients through various times of remission status. T-SNE analysis of the 9 miRNAs of the patients in remission and among healthy patients showed separation between the majority of samples (Fig. 5A). Of note, there were 2 serum samples collected from DLBCL patients that at the time of blood draw who were thought to be in remission, however several months later, the patients relapsed. When only considering the 5 significant miRNAs from the signature determined from the ROC analysis (Fig. 5B), we observed similar better separation between healthy individuals and DLBCL patients <24 months versus ≥24 months of remission (Fig. 5C).

We observed two distinct hierarchical clusters when comparing healthy and remission patients combined together. One cluster included 25 remission patients that grouped closely with healthy subjects (blue cluster tree in Fig. 5D). The other 26 remission patients clustered together (orange cluster tree in Fig. 5D) showing an increase in miRNA levels, while 10 of these patients had high levels present. Interestingly, 9 of those 10 remission patients were diagnosed with *MYC* positive DLBCL. Lastly, one patient thought to be in remission at time of sample collection, but subsequently relapsed within 6 months, showed the highest amounts of circulating miRNAs in the serum (Fig. 5D).

### Network analysis with the circulating 5 multi-miRNA signature

**Figure 6:**
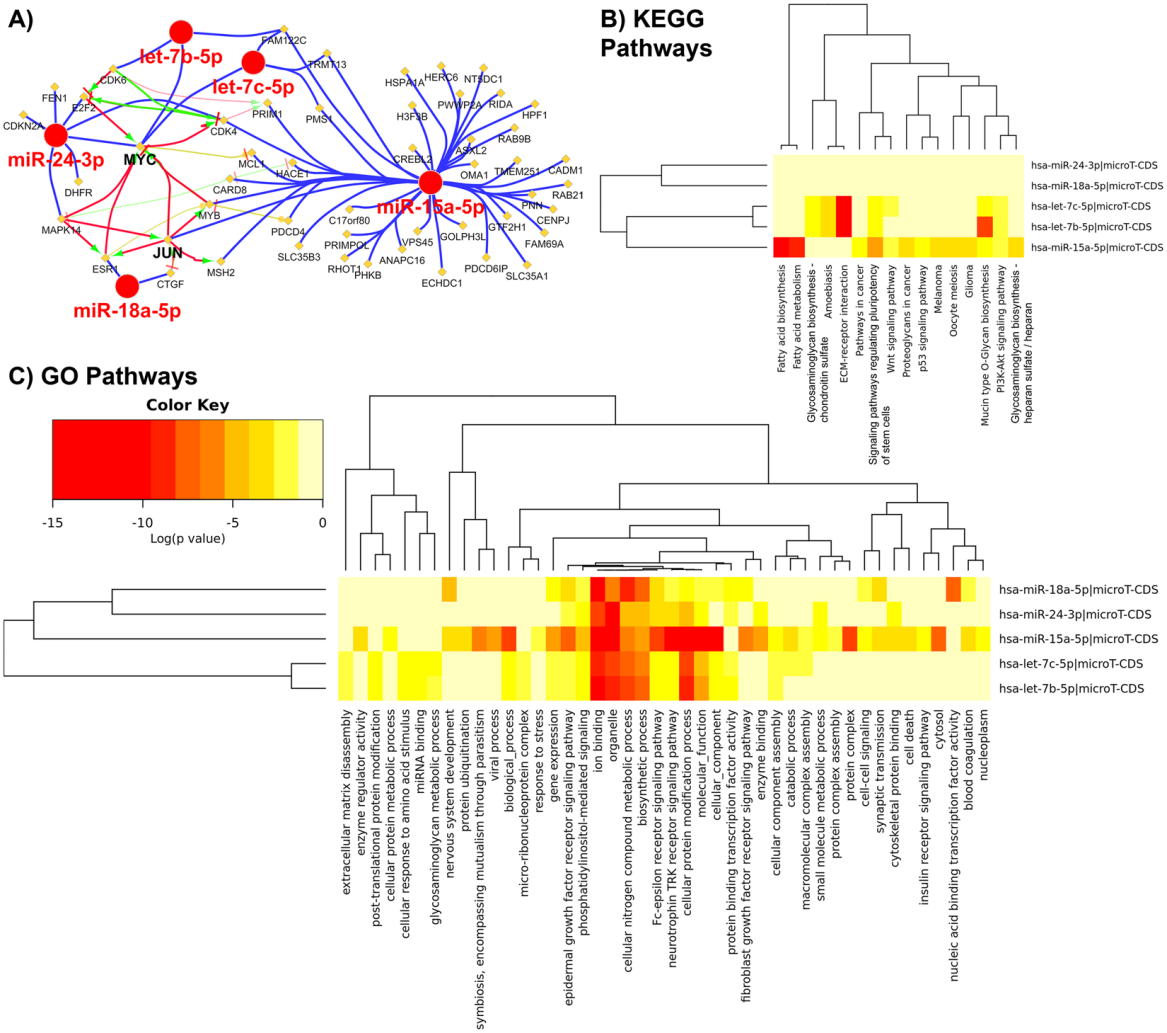
Predicted genes and biological functions to be the most impacted by the 5 miRNAs included in the signature. (**A**) The five miRNAs selected for the signature (Supplemental Tables S4 and S5) were used to predict which genes will be the most regulated by these miRNAs determined using CluePedia Cytoscape plugin^50^. The miRNA-mRNA interactions were determined from three different databases. MYC is the most regulated by these miRNAs with JUN be the second most regulated. (**B**,**C**) The impact of the five miRNAs on the KEGG and Gene Ontology (GO) biological pathways predicted with DIANA microT-CDS tool^24^. Heatmap representation of the pathways and the significance (determined from log(p-values)) with each miRNA with red indicating the highest level of significance and yellow the lowest level of significance.

*In silico* predictions showed that *MYC* was the most regulated gene by these 5 miRNAs (let-7b, let-7c, miR-18a, miR-24, and miR-15a) with *JUN* being the second-most regulated (Fig. 6A). *JUN* has been shown to be activated and contribute to DLBCL growth^22^; and we recently reported a strong association between age and *JUN* expression in DLBCL^23^. We further predicted the biological functional impact of these 5 miRNAs utilizing DIANA Tools microT-CDS miRNA functional predictions^24,25^. The KEGG pathway microT-CDS analysis indicated these miRNAs dysregulate functions that are closely related to DLBCL progression (Fig. 6B). Pathways related to fatty acid biosynthesis and metabolism are shown to be regulated. We showed before that fatty acid synthase is involved with DLBCL progression in part by inducing PI3K-Akt-S6Kinase signaling to enhance interactions a USP11-eIF4B complex^26^. Interestingly, PI3K-Akt signaling is another pathway that is regulated by these 5 miRNAs. The role of heparan-sulphate glycosaminoglycans has been shown to be heavily involved in cancer development and specifically lymphomas^27^. Both WNT signaling pathways^28^ and p53 signaling pathways have been shown to be heavily involved with DLBCL.

With the Gene Ontology (GO) pathway microT-CDS analysis, we determined biological functions involved with the 5 miRNAs (Fig. 6C) to illustrate relevance to DLBCL progression. The top 4 significant GO pathways that the 5 miRNAs are predicted to regulate are relevant to DLBCL progression. For example, the GO cellular nitrogen compound metabolic process involves pathways related to chemical reactions with nitrogenous compounds such as mTOR signaling through involvement of various metabolic processes (i.e. amino acid metabolic process). It has been reported that this process is widely involved with DLBCL metabolic reprogramming^29^. Related ion binding pathways, specifically genes related to ion channels and transporters, as have been associated with DLBCL development and relapse in patients^30^. Other pathways such as neurotrophins and Trk signaling^31^ and Fc epsilon receptor signaling^32^ have been shown to be involved in DLBCL resistance to therapeutic drugs.

## Discussion

There remains a significant need for sensitive, robust, and reliable technologies to prospectively detect and characterize cancer activity. This is particularly salient in lymphoma where there are often not circulating tumor cells present in the blood with currently available methods (e.g., flow cytometry). miRNAs are small non-coding RNAs impacting post-transcriptional gene expression that have shown to have a major impact on lymphoma biology as well as associated therapeutic potential^10,13,19,23^. Furthermore, these miRNAs are in the circulating serum, are stable, and are resistant to degradation^15,19^. Leveraging findings from an initial transgenic lymphoma knockout model^19^, we investigated for the presence of circulating miRNAs in human DLBCL cells, DLBCL PDX models, and sera from DLBCL patients. We confirmed that these circulating miRNAs were readily detectable and highly elevated in DLBCL PDX models. Additionally, there were significantly increased circulating levels of the miRNA signature in the sera of DLBCL patients, especially a 5 multi-RNA signature (i.e., let-7b, let-7c, miR-18a, miR-24, and miR-15a). Particular miRNAs were also associated with patient stage and the presence of MYC overexpression or rearrangement in patients with DLBCL. Furthermore, circulating miRNAs appeared to distinguish DLBCL patients in remission from healthy controls based on a novel 5 multi-miRNA signature.

The majority of miRNA research and identification of miRNAs associated with B-cell lymphomas have utilized tumor biopsy samples to compare lymphoma with nonmalignant tissues^33–35^. From these studies, there have been several miRNAs consistently identified to be associated with DLBCL that include miR-155^3,33–35^, miR-21^10,12,33–35^, miR-15a/miR-16^10,33,34^, miR-29c^10,33^, miR-34a^33–35^, miR-125b^33,36^, miR-130a^33,36^, miR-17–92^33–35^, and miR-150^34^. Each of these prior studies focused on *one or two miRNAs* and linked expression to aspects of DLBCL biology or behavior. For example, increased levels of miR-155 in patient DLBCLs confers a poor prognosis^3,34,35^. In fact, the first publication on the detection of DLBCL associated circulating miRNAs only focused on three miRNAs drawn from existing knowledge associated with DLBCL and miRNAs, with miR-155 as one of the miRNAs studied^18^. Other miRNAs, such as miR-21 have been shown to be significantly elevated in DLBCL patients^37^, have specific sensitivity to treatment^38^, and using *in vivo* models are determined to impact tumor dynamics^39^.

The complexity of miRNA interactions and the ability to identify the potential drivers for DLBCL is challenging. The majority of research of miRNAs in lymphoma has compared DLBCL patients directly to healthy controls. These studies have revealed thousands of miRNAs that are often dysregulated between groups adding complexity to this determination. To overcome these issues and to identify a multi-miRNA signature associated with DLBCL, we utilized a systems biology approach. This approach was based on the concept that the there are multiple important miRNAs present and that the connectivity and interactions with transcriptomic expression may dictate DLBCL progression.

In addition, an optimal method to robustly and precisely quantify miRNA via ddPCR was employed for these studies^6,40^. Utilizing this technology, we previously identified 10 miRNAs associated with DLBCL via a transgenic murine model, which produces spontaneous *MYC*-related DLBCL^19^. From these miRNAs, 9 of 10 were robustly detectable in the serum of DLBCL patients, with 5 being highly prominent (miR-18a, miR-15a, let-7c, let-7b, and miR-24). miR-15a and miR-18a, which have been shown to be part of the miR-17-92 cluster, were previously associated with DLBCL^33–35^. For most cancers, positive upregulation of the let-7 family has been shown to suppress cancer growth^41,42^. The majority of these studies were studying let-7 as the individual impact it has on cancer rather than as part of a larger group of miRNAs acting as a signature associated with that cancer type. Which is why specific members of the let-7 family, such as let-7b, have been shown for the same tumor to be both a tumor promoter^43^ and tumor suppressor^44^. We identified that higher levels of circulating let-7b were associated with stage III-IV versus I-II disease in DLBCL and that higher levels of miR-27a and miR-24 were associated with *MYC* rearrangement. Furthermore, higher levels of miR-18a were associated with Myc protein overexpression by IHC as well as having received prior therapy.

We also analyzed the circulating miRNA signature here for associations with remission status. We discovered that the 5-miRNA subset appeared to distinguish remission time points for DLBCL patients (Fig. 5B,C). We confirmed that while miRNA levels for the majority of DLBCL patients in remission did not completely return to levels in healthy controls, there was a prominent decrease for the majority of patients to the levels of healthy subjects (Fig. 5D). It is possible that the persistently higher levels of miRNAs among some DLBCL patients in remission may be associated with the potential for relapse. There are emerging studies in cancer elucidating the importance of circulating miRNA changes in the blood of patients^45,46^. This includes Frères *et al*. who recently identified that circulating miRNAs in patients with breast cancer could serve as a potential biomarker for chemotherapy-related cardiac dysfunction^47^. They showed that although there is a decrease in the miRNAs (similar to what we have observed for DLBCL patients), the amounts of the circulating miRNAs never return to normal/healthy levels.

In conclusion, we identified that a circulating 5 multi-miRNA signature was readily and prominently detected in human DLBCL cell lines, DLBCL PDX models, and from the sera of DLBCL patients. Prospective clinical studies are warranted to validate these findings, including differentiating varied clinical subsets of patients and pathologic differences in DLBCL. Confirmatory studies in larger patient populations will also be needed to prove the reliability of utilizing ddPCR testing of circulating miRNAs as a minimally invasive biomarker in DLBCL as well as to potentially examine for early relapse. Finally, these miRNAs may also be potentially harnessed as a potential novel therapeutic platform for the targeted treatment of DLBCL.

## Materials and Methods

### Patient derived xenograft (PDX) model for lymphoma and collection of blood

We utilized a previously established multiple *MYC-*rearranged and -unrearranged DLBCL PDX models from tumors of patients with DLBCL^21^. Briefly, de-identified patient samples were obtained with informed consent and xenografted under Dana-Farber/Harvard Cancer Center Institutional Review Board (IRB)-approved protocol #13-351. Nod.Cg-*Prkdc*^*SCID*^*IL2Rγ* < tm1Wjl > /SzJ (NSG) mice were purchased from Jackson Laboratories (Bar Harbor, ME) and were implanted with the PDX lines DFBL-74251, DFBL-69487, DFBL-75549, and DFBL-20954. The PDX mouse models were handled in accordance to the guidelines and regulations with Dana-Farber Cancer Institute’s Institutional Animal Care and Use Committee (IACUC). All experimental protocols were approved by Dana-Farber Cancer Institute’s IACUC with approval protocol #13–034. Following tumor transplantation, 200μL of blood was drawn via submandibular bleeding from the PDX mice to analyze circulating miRNA. The blood was allowed to clot for 30 min at room temperature and then centrifuged at 1000 × g for 10 min. The serum was removed into a new tube and stored at −80 °C until use.

### Lymphoma cell lines utilized for miRNA quantification

DLBCL commercial cell lines SUDHL4 and SUDHL10 were purchased from American Type Culture Collection (Manassas, VA, USA) and used for miRNA quantification. These cells were maintained in RPMI-1640 medium supplemented with 10% Fetal Bovine Serum and 1% antibiotic-antimycotic solution (Corning Cellgro, Manassas, VA, USA). A primary human lymphoma cell line (EL-2) were isolated from discarded tissue of a patient with DLBCL obtained through the Tufts Tumor Repository under an IRB-approved protocol, as described previously^48^.

### Blood collection from DLBCL patients and healthy controls

Blood from both DLBCL patients and healthy donors was collected to assess miRNA levels. We collected 4 mL of blood from 86 patients with DLBCL with the following conditions: pre-treatment with first-line therapy (n = 11), progression after treatment (n = 7), during treatment (n = 16), and in clinical complete remission (n = 52). In addition, we collected 4 mL of blood from healthy non-tumor bearing individuals for controls (n = 17). All blood drawn from patients and healthy donors were done by standard venipuncture sampling. The same techniques used with the PDX mice were used to isolate and store the serum from these samples. De-identified patient samples were obtained with informed consent under the approved Dana-Farber/Harvard Cancer Center Institutional Review Board (IRB) (approved protocol #13-351) and Tufts Medical Center/Tufts Health Sciences Campus Institutional Review Board (approved protocols #9429 and #12466). This study was performed in accordance with the principles of the Declaration of Helsinki. Informed consent from all subjects were obtained and all subjects were over 18 years of age.

### miRNA Isolation

Isolation of circulating miRNAs from serum directly from cell lines was done using the QIAgen miRNeasy Mini Kit (QIAgen, CA) as previously reported^19^. Concentration and quality of miRNAs was quantified by the Eppendorf Biophotometer 6131 spectrophotometer (Eppendorf, Hauppauge, NY).

### cDNA generation and Droplet Digital PCR

A schematic of the overall methodology used to quantify the miRNAs is shown in Supplemental Fig. S1. From the isolated miRNA, cDNA was first generated using the miRCURY LNA^TM^ Universal RT microRNA PCR Universal cDNA Synthesis Kit II (Exiqon, Woburn, MA) using a concentration of 5 ng/μl for the miRNA per sample. The miRNA was quantified using the Bio-Rad QX200^TM^ Droplet Digital PCR system (ddPCR) (Bio-Rad, Hercules, CA). A 1:20 dilution of the generated cDNA was used with the ddPCR EvaGreen Supermix (Bio-Rad). The following miRCURY LNA^TM^ Universal RT microRNA PCR LNA^TM^ PCR primer sets (Exiqon) were used with the EvaGreen Supermix: hsa-let-7c-5p, hsa-let-7b-5p, mmu-miR-497-5p, hsa-miR-130a-3p, hsa-miR-18a-5p, hsa-miR-24-3p, hsa-miR-15a-5p, hsa-miR-27a-3p, hsa-miR-10b-5p, and mmu-miR-155-5p. These miRNAs are highly conserved between mice and humans and we have tested that all primers utilized for this manuscript will identify the specific miRNAs in both species. Droplets were generated using the QX200™ Automated Droplet Generator (Bio-Rad). The plates were sealed with the PX1^TM^ PCR Plate Sealer (Bio-Rad).

With the C1000 Touch™ Thermal Cycler with 96–Deep Well Reaction Module (Bio-Rad) the following PCR reaction was used for all the primers except for hsa-miR-10b-5p and mmu-miR-155-5p: 1 cycle 95 °C for 5 min, 40 cycles of 95 °C for 30 sec and 58 °C for 1 min (annealing temperature), 1 cycle of 4 °C for 5 min, and 1 cycle of 90 °C for 5 min. Not all miRNA primers sets for ddPCR will have the same annealing temperature, so optimizing the annealing temperature is required for each primer set. For hsa-miR-10b-5p and mmu-miR-155-5p primer sets the optimal annealing temperatures were 54 °C and 52 °C respectively. The QX200™ Droplet Digital™ PCR System (Bio-Rad) quantified the amount of miRNA for each primer set per sample. QuantaSoft software (Bio-Rad) generated the data for each primer set and sample. The same threshold setting was used for all samples per primer set. The concentration (miRNA copies/μl) value generated by QuantaSoft was converted to miRNA copies/ng of serum. An example of the output for the software and the comparison to positive and negative droplets are shown in Supplemental Fig. S1.

### *In silico* predictions of genes from miRNAs

Through the use of a Cytoscape^49^ plug-in called ClueGo/CluePedia^50^, we were able to predict genes targeted by the miRNAs determined. This involved entering all miRNAs in ClueGo and allowing the software to determine the top 50 genes that were significantly regulated and connected to the miRNAs. The predictions only reflect the functions that will be regulated by the miRNAs and do not show whether the function will be activated or inhibited. We also predicted the impact of the miRNAs on overall pathways by utilizing DIANA microT-CDS tool which allows for pathway discovery from targeted miRNAs^24,25^.

### Statistics

For continuous measures, a Wilcoxon rank-sum test or a Kruskal-Wallis was used for comparison between or among groups. For categorical comparisons, a Fisher exact test was performed. Unsupervised principal components analysis (PCA) was used to determine trends and clustering of miRNA levels by patient’s disease status. Receiver operator curves (ROCs) were constructed to compare healthy controls versus the on-treatment/progression samples for each miRNA. The area under the curve (AUC) was also calculated. The optimal cutpoint for the comparison of on-treatment/progression samples to healthy samples was determined using the SAS macro %ROCPLOT and was based on the Youden index. To develop a final signature, the package rpart (R version 3.5.0) was used for recursive partitioning analysis, with similar cutpoints obtained also from AUC analysis comparing on-treatment/progression samples to healthy controls. Using these cutpoints, remission samples were classified and the correct classification rate, sensitivity, specificity were calculated. ROCs were also constructed comparing remission samples to healthy samples. Hierarchical clustering analysis was performed using Euclidean distance with complete linkage to produce heatmaps. *P*-values reported are two-sided and adjusted by using a < 0.01 significance level for association with clinical characteristics.

## Supplementary information

Supplementary Material
