# Supplementary material for: Identification of Circulating Serum Multi-MicroRNA Signatures in Human DLBCL Models
Source: Sci Rep. 2019 Nov 20;9:17161. doi: 10.1038/s41598-019-52985-x (PMC6868195; doi:10.1038/s41598-019-52985-x)
Supplement: Supplementary file 1 — Supplementary Material [file 41598_2019_52985_MOESM1_ESM.docx]

**Supplementary Material for:**

**Identification of Circulating Serum Multi-MicroRNA Signatures in Human DLBCL Models**

Afshin Beheshti, PhD^1,2,5*^, Kristen Stevenson, MS^3,4^, Charles Vanderburg, PhD^4,5^, Dashnamoorthy Ravi, PhD^2^, J. Tyson McDonald, PhD^6^, Amanda L. Christie^3^, Kay Shigemori^3^, Hallie Jester^3^, David M. Weinstock, MD^3,4^, and Andrew M. Evens, DO, MSc^2^

^1^WYLE, NASA Ames Research Center, Moffett Field CA, USA
^2^Division of Blood Disorders, Rutgers Cancer Institute of New Jersey, New Brunswick, NJ

^3^Department of Medical Oncology, Dana-Farber Cancer Institute, Boston, MA, USA

^4^Harvard Medical School, Boston, MA, USA

^5^Stanley Center for Psychiatric Research, Broad Institute of MIT and Harvard, Cambridge, MA, USA

^6^Cancer Research Center and Department of Physics, Hampton University, Virginia, USA

**Supplemental Table S1.** Fold-Change differences between the mean miRNA amounts present in the serum of the PDX models compared to the miRNA amounts present in the corresponding cell line.

| PDX | miR-15a | miR-18a | miR-24 | miR-27a | let-7b | let-7c | miR-130a | miR-10b | miR-155 |
| --- | --- | --- | --- | --- | --- | --- | --- | --- | --- |
| DFBL-74251 | 5.77 | 0.59 | 0.47 | 0.63 | 2.20 | 0.57 | 27.37 | 1.62 | 0.02 |
| DFBL-20954 | 2.15 | 0.51 | 1.46 | 3.43 | 1.67 | 0.39 | 13.70 | 0.35 | 0.14 |
| DFBL-69487 | 0.21 | 0.06 | 1.63 | 1.57 | 3.48 | 0.23 | 18.08 | 1.54 | 0.01 |
| DFBL-75549 | 754.55 | 17.83 | 3111.67 | 235.00 | 96.36 | 19.39 | 76.00 | 13.71 | 0.86 |

**Supplemental Table S2. Patient Characteristics**

|  | DLBCL  N (%) | Healthy Controls  N (%) |
| --- | --- | --- |
| Total, N | 86 | 17 |
| Sex |  |  |
| Female | 26 (30) | 11 (65) |
| Male | 59 (69) | 6 (35) |
| Unknown | 1 (1) | 0 (0) |
| Age, median (range) | 64 (21, 87) | 52 (29, 70) |
| 20 – 49 yrs | 12 (14) | 4 (24) |
| 50 – 59 yrs | 21 (25) | 12 (71) |
| 60 – 69 yrs | 22 (26) | 0 (0) |
| 70 – 79 yrs | 24 (28) | 1 (6) |
| ≥80 yrs | 6 (7) | 0 (0) |
| Unknown | 1 (1) | 0 (0) |
| Stage at Dx |  |  |
| 1 | 16 (19) |  |
| 2 | 8 (9) |  |
| 3 | 12 (14) |  |
| 4 | 22 (26) |  |
| Unknown | 28 (33) |  |
| LDH, median (range) (n=73, DLBCL only) | 171 (103, 777) |  |
| MYC FISH rearrangement |  |  |
| Present | 13 (16) |  |
| Absent | 41 (52) |  |
| Unknown | 25 (32) |  |
| MYC Positive (IHC) |  |  |
| Negative or <=10% | 15 (17) |  |
| >10 - 30% | 19 (22) |  |
| 40% to < 60% | 15 (17) |  |
| 60% to >90% | 11 (13) |  |
| Unknown | 26 (30) |  |
| BCL2 Positive (IHC) | 52 (60) |  |
| Negative | 11 (13) |  |
| Unknown | 23 (27) |  |
| BCL6 Positive (IHC) | 59 (69) |  |
| Negative | 3 (3) |  |
| Unknown | 24 (28) |  |
| Any Prior AutoSCT | 22 (26) |  |
| Any Prior AlloSCT | 6 (7) |  |
| Any Prior CAR-T | 5 (6) |  |
| Number of Prior Therapies, median (range) | 1 (0, 9) |  |
| 0 | 11 (13) |  |
| 1 | 37 (43) |  |
| 2 | 5 (6) |  |
| 3 | 13 (15) |  |
| ≥4 | 20 (24) |  |
| Last Tx Prior to Sample |  |  |
| Treatment Naive | 11 (13) |  |
| RCHOP +/- Other | 32 (38) |  |
| R-EPOCH | 5 (6) |  |
| RICE | 3 (4) |  |
| Pembro/Nivo | 4 (5) |  |
| Allo SCT | 6 (7) |  |
| Auto SCT | 10 (12) |  |
| CAR-T | 5 (6) |  |
| IFRT/ISR | 2 (2) |  |
| Other | 6 (7) |  |
| Unknown | 2 (2) |  |
| Months in Remission at time of Sample, median (range)† | 19 (0, 93) |  |
| Disease Status at Time of Sample |  |  |
| Remission |  |  |
| <24 mos. | 31 (36) |  |
| ≥24 mos. | 21 (24) |  |
| Treatment Naive | 11 (13) |  |
| On Treatment | 16 (19) |  |
| Progression | 7 (8) |  |

†One case had remission confirmed 17 days after sample is classified as a remission at the time of sample.

**Supplemental Table S3A.** Summary Statistics of the Concentration Copies/ng for Healthy Controls.

| **miRNA** | **cat** | **N** | **Min** | **Median** | **Max** | **Mean** | **Std Dev** | **LCL 95% for Mean** | **UCL 95%  for Mean** |
| --- | --- | --- | --- | --- | --- | --- | --- | --- | --- |
| miR-27a-3p | healthy | 17 | 0.24 | 1.48 | 3.44 | 1.63 | 1.02 | 1.11 | 2.16 |
| miR-24-3p | healthy | 17 | 0.00 | 1.36 | 4.80 | 1.78 | 1.40 | 1.06 | 2.50 |
| miR-18a-5p | healthy | 17 | 0.00 | 0.44 | 2.08 | 0.65 | 0.59 | 0.35 | 0.95 |
| miR-15a-5p | healthy | 17 | 0.20 | 1.36 | 6.40 | 1.71 | 1.65 | 0.86 | 2.56 |
| miR-155-5p | healthy | 17 | 0.00 | 0.84 | 4.80 | 1.04 | 1.09 | 0.49 | 1.60 |
| miR-130a-3p | healthy | 17 | 0.00 | 0.24 | 1.96 | 0.43 | 0.54 | 0.15 | 0.71 |
| miR-10b-5p | healthy | 17 | 0.00 | 0.48 | 4.80 | 0.89 | 1.15 | 0.30 | 1.48 |
| let-7c-5p | healthy | 17 | 3.96 | 6.00 | 12.40 | 6.80 | 2.48 | 5.52 | 8.07 |
| let-7b-5p | healthy | 17 | 0.24 | 0.96 | 10.00 | 2.34 | 2.49 | 1.05 | 3.62 |

LCL = lower confidence limit UCL=upper confidence limit

**Supplemental Table S3B.** Summary Statistics of the Concentration Copies/ng for Healthy Controls using a log_2_(x+1) transformation.

| **miRNA** | **cat** | **N** | **Min** | **Median** | **Max** | **Mean** | **Std Dev** | **LCL 95% for Mean** | **UCL 95%  for Mean** |
| --- | --- | --- | --- | --- | --- | --- | --- | --- | --- |
| miR-27a-3p | healthy | 17 | 0.31 | 1.31 | 2.15 | 1.29 | 0.57 | 1.00 | 1.59 |
| miR-24-3p | healthy | 17 | 0.00 | 1.24 | 2.54 | 1.31 | 0.71 | 0.95 | 1.68 |
| miR-18a-5p | healthy | 17 | 0.00 | 0.53 | 1.62 | 0.65 | 0.45 | 0.42 | 0.88 |
| miR-15a-5p | healthy | 17 | 0.26 | 1.24 | 2.89 | 1.23 | 0.76 | 0.84 | 1.62 |
| miR-155-5p | healthy | 17 | 0.00 | 0.88 | 2.54 | 0.90 | 0.58 | 0.61 | 1.20 |
| miR-130a-3p | healthy | 17 | 0.00 | 0.31 | 1.57 | 0.44 | 0.47 | 0.19 | 0.68 |
| miR-10b-5p | healthy | 17 | 0.00 | 0.57 | 2.54 | 0.77 | 0.61 | 0.45 | 1.08 |
| let-7c-5p | healthy | 17 | 2.31 | 2.81 | 3.74 | 2.91 | 0.40 | 2.70 | 3.11 |
| let-7b-5p | healthy | 17 | 0.31 | 0.97 | 3.46 | 1.46 | 0.86 | 1.02 | 1.91 |

**Supplemental Table S4. Classification of Remission Samples based on Cutpoints Determined by Healthy vs. On-Treatment/Progression Samples.**

| **miRNAs** | **ROC**  **Cutpoint** | **Youden Index** | **DLBCL Remission**  **(n=52)**  **n (%)** | **Healthy**  **(n=17)**  **n (%)** | **Sensitivity**  **(%)** | **Specificity (%)** | **Correct Classification**  **(%)** |
| --- | --- | --- | --- | --- | --- | --- | --- |
| **miR-27a** | ≥ 9.2 | 0.48 | 32 (62) | 0 (0) | 0.62 | 1.00 | 0.46 |
|  | < 9.2 |  | 20 (38) | 17 (100) |  |  |  |
| **miR-24** | ≥ 6.0 | 0.83 | 44 (85) | 0 (0) | 0.85 | 1.00 | 0.88 |
|  | < 6.0 |  | 8 (15) | 17 (100) |  |  |  |
| **miR-18a** | ≥ 2.1 | 0.96 | 38 (73) | 0 (0) | 0.73 | 1.00 | 0.80 |
|  | < 2.1 |  | 14 (27) | 17 (100) |  |  |  |
| **miR-15a** | ≥ 4.4 | 0.81 | 39 (75) | 1 (6) | 0.75 | 0.94 | 0.80 |
|  | < 4.4 |  | 13 (25) | 16 (94) |  |  |  |
| **miR-155** | ≥ 1.7 | 0.40 | 23 (44) | 2 (12) | 0.44 | 0.88 | 0.55 |
|  | < 1.7 |  | 29 (56) | 15 (88) |  |  |  |
| **miR-130a** | ≥ 1.3 | 0.77 | 40 (77) | 1 (6) | 0.77 | 0.94 | 0.81 |
|  | < 1.3 |  | 12 (23) | 16 (94) |  |  |  |
| **miR-10b** | ≥ 0.5 | 0.43 | 33 (63) | 6 (35) | 0.63 | 0.65 | 0.64 |
|  | < 0.5 |  | 19 (37) | 11 (65) |  |  |  |
| **let-7c** | ≥ 14.0 | 0.96 | 39 (75) | 0 (0) | 0.75 | 1.00 | 0.81 |
|  | < 14.0 |  | 13 (25) | 17 (100) |  |  |  |
| **let-7b** | ≥ 18.8 | 0.83 | 41 (79) | 0 (0) | 0.79 | 1.00 | 0.84 |
|  | < 18.8 |  | 11 (21) | 17 (100) |  |  |  |
| **Signature*** | * |  | 47 (90) | 1 (6) | 0.90 | 0.94 | 0.91 |
|  |  |  | 5 (10) | 16 (94) |  |  |  |

ROC=receiver operating curve

*Cut-points: miR-24 < 5.4 and miR-18a <2.1 and miR-15a < 4.2 and let-7c < 13.2 and let-7b < 14.4 obtained from recursive partitioning analysis comparing Healthy vs. On-Treatment/Progression Samples.


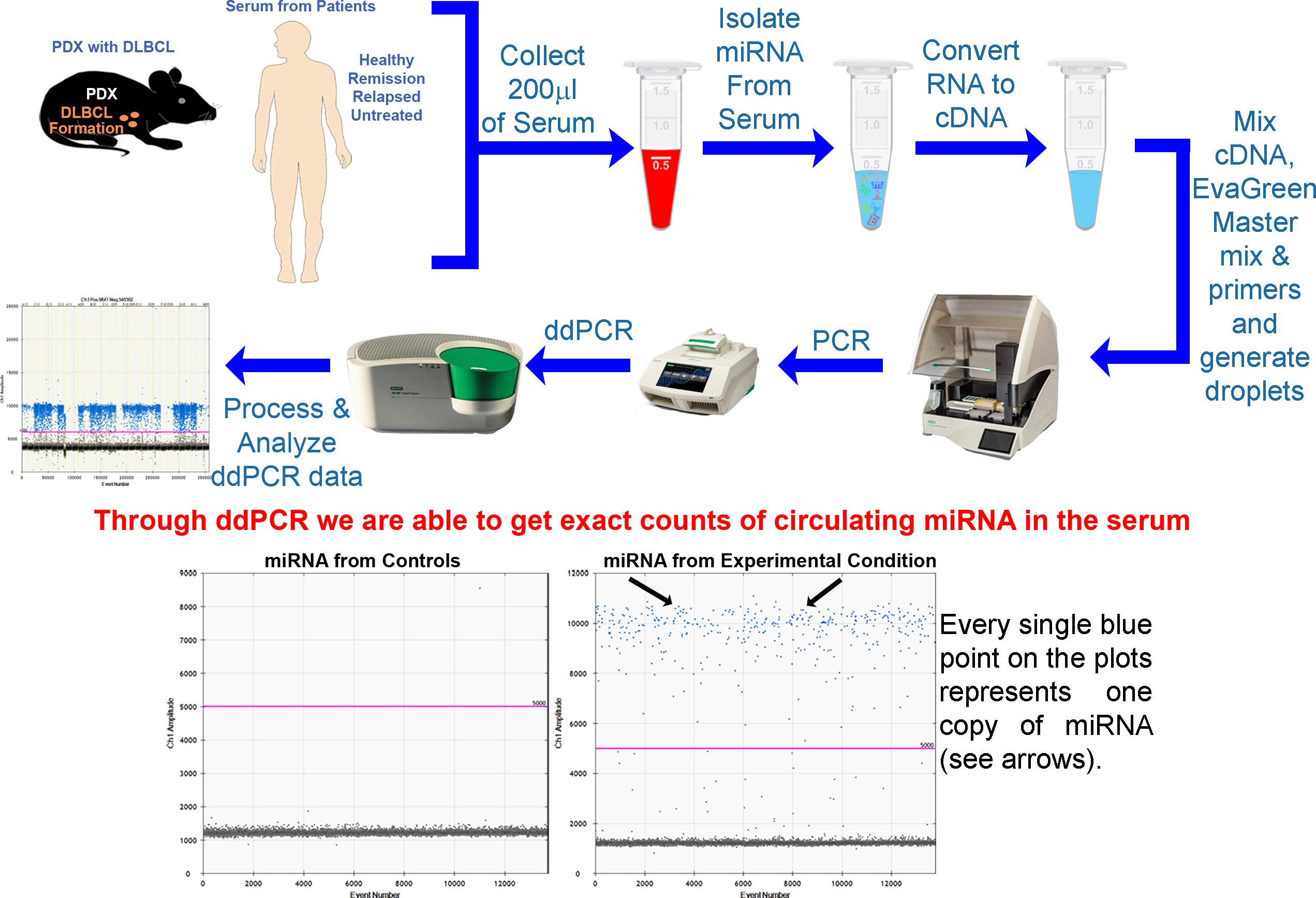


**Supplemental Figure 1.** **Schematic of the methods used to obtain the data.** Serum from either mice (200 µl) or patients (4 mL) were collected and miRNA was isolated. The miRNA was converted to cDNA and mixed with the EvaGreen Master Mix and miRNA primers for droplet generation and ddPCR. The final step was placing the plate after PCR into the plate reader to quantify the number miRNA copies/µl for each sample. The negative droplets were observed on the bottom of the dot plots while the positive droplets indicate each copy of a miRNA detected in the sample. A consistent threshold is set for quantification to provide the normalized miRNA expression.
